# Multiple roles of DNA2 nuclease/helicase in DNA metabolism, genome stability and human diseases

**DOI:** 10.1093/nar/gkz1101

**Published:** 2019-11-22

**Authors:** Li Zheng, Yuan Meng, Judith L Campbell, Binghui Shen

**Affiliations:** 1 Department of Cancer Genetics and Epigenetics, Beckman Research Institute of City of Hope, 1500 East Duarte Road, Duarte, CA 91010, USA; 2 Divisions of Chemistry and Chemical Engineering and Biology and Biological Engineering, California Institute of Technology, Pasadena, CA 91125, USA

## Abstract

DNA2 nuclease/helicase is a structure-specific nuclease, 5′-to-3′ helicase, and DNA-dependent ATPase. It is involved in multiple DNA metabolic pathways, including Okazaki fragment maturation, replication of ‘difficult-to-replicate’ DNA regions, end resection, stalled replication fork processing, and mitochondrial genome maintenance. The participation of DNA2 in these different pathways is regulated by its interactions with distinct groups of DNA replication and repair proteins and by post-translational modifications. These regulatory mechanisms induce its recruitment to specific DNA replication or repair complexes, such as DNA replication and end resection machinery, and stimulate its efficient cleavage of various structures, for example, to remove RNA primers or to produce 3′ overhangs at telomeres or double-strand breaks. Through these versatile activities at replication forks and DNA damage sites, DNA2 functions as both a tumor suppressor and promoter. In normal cells, it suppresses tumorigenesis by maintaining the genomic integrity. Thus, DNA2 mutations or functional deficiency may lead to cancer initiation. However, DNA2 may also function as a tumor promoter, supporting cancer cell survival by counteracting replication stress. Therefore, it may serve as an ideal target to sensitize advanced DNA2-overexpressing cancers to current chemo- and radiotherapy regimens.

## INTRODUCTION

Maintaining the integrity of the genome depends on faithful DNA replication and proper repair of DNA damage. Various DNA intermediates are formed during these DNA metabolic processes, and they must be efficiently and properly processed to avoid severe genomic instability. The most frequently occurring intermediates are Okazaki fragments, which are formed during lagging strand DNA synthesis (1). It is estimated that millions of Okazaki fragments are generated per mammalian cell cycle (2). Each Okazaki fragment contains an RNA–DNA primer, synthesized by the Pol α (DNA polymerase subunit alpha)/primase complex, at its 5′ end. The RNA portion of the primer must be removed so that the Okazaki fragments can be joined to form intact lagging strand DNA (2). In the case that Pol α introduces errors, the Pol α synthesized DNA may be removed via nucleotytic editing mechanisms before joining (2). Meanwhile, DNA molecules frequently encounter DNA-damaging insults that cause various lesions, including base damage, inter-strand cross-links, DNA single-strand breaks (SSBs) and double-strand breaks (DSBs). Such DNA lesions and the intermediates that form during their metabolism must be repaired to avoid DNA mutations, deletions, insertions and translocations (3). For example, other commonly generated replication intermediates are stalled replication forks, which can arise due to both endogenous or exogenous replication barriers, such as stable secondary structures on the DNA template, protein–DNA complexes and DNA lesions (4–6). Stalled replication forks may be transformed into regressed forks to promote fork restart; however, these structures are potentially deleterious and must be properly processed to restart DNA replication without introducing errors (4). Specific nuclease and helicase complexes recognize and are required to process different subsets of DNA intermediates, including Okazaki fragments and stalled replication forks. Helicases can unwind and transform the intermediates, whereas nuclease complexes cleave phosphodiester bonds, independent of DNA sequence, to ultimately produce DNA structures appropriate for ligation, continuous DNA replication or recombination.

DNA2 nuclease/helicase, an enzyme conserved in eukaryotic organisms, is critical for the metabolism of several DNA intermediates (Table 1). DNA2 was originally discovered through the characterization of a temperature-sensitive and DNA replication-defective mutant strain of *Saccharomyces cerevisiae* (yeast), namely the *dna2* mutant strain (7). *Saccharomyces cerevisiae dna2* mutants also emerged in a genetic screen for strains that require overexpression of the protein kinase Tor1p for viability (8) and as a gene synthetic lethal with *ctf4* mutations (9). The *S. cerevisiae DNA2* (*sc*DNA2) gene encodes a 172-kD (1522-amino acid) protein, which has nuclease, 5′-to-3′ DNA helicase, and DNA-dependent ATPase activities. It has a PD-(D/E)XK nuclease superfamily motif at its center and an ATP-dependent SF1B helicase family motor domain at its C-terminus (7,10). Additional studies also identified a regulatory N-terminal domain (11,12) that interacts with the nuclease domain and inhibits its endonuclease activity. Thus, the removal of this regulatory domain using proteolysis greatly stimulates the nuclease activity of *sc*DNA2. In addition, an iron–sulfur (Fe–S) cluster motif was identified within the nuclease domain of *sc*DNA2 (13). Surprisingly, mutations that disrupt the Fe–S cluster motif abolish not only the nuclease but also the helicase activities of *sc*DNA2, suggesting that the Fe–S cluster plays a role in coupling them (13). The *sc*DNA2 protein also has three types of classical nuclear localization signals (NLSs) that direct its migration into the nucleus (14).

**Table 1.** Summary of DNA2 substrates and activities in various pathways across organisms

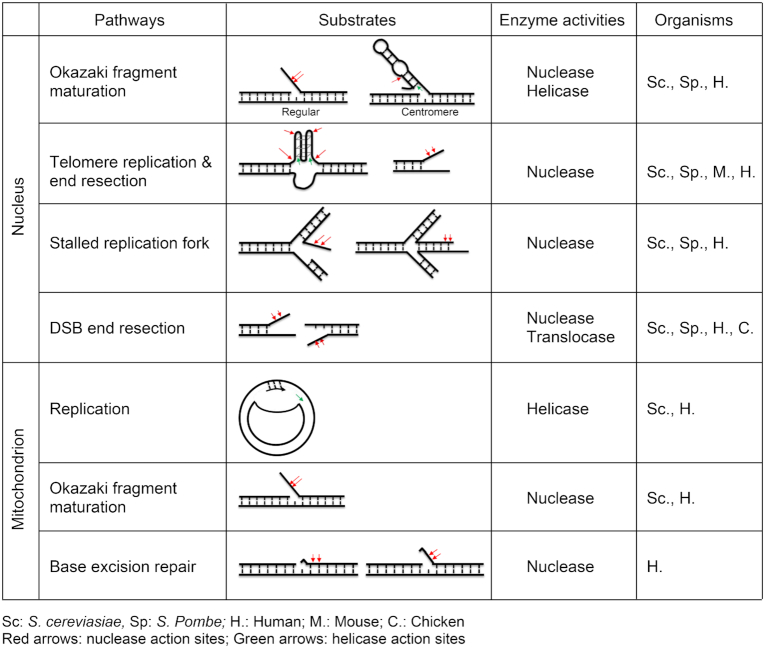

*sc*DNA2 orthologs were subsequently identified in *Schizosaccharomes pombe* (fission yeast) (15), *Caenorhabditis elegans* (16), *Xenopus laevis* (17) and mammals (18–20). Like *sc*DNA2, they all have a core nuclease domain, ATPase/helicase domains, and an Fe–S cluster motif. Interestingly, throughout evolution, DNA2 proteins have gradually lost NLSs (14). Whereas Dna2 proteins in single-cell organisms, such as *S. cerevisiae* and *S. pombe*, have all three types of classical NLSs, their orthologs in *Arabidopsis*, *C. elegans*, *Drosophila*, puffer fish, and frogs, have only one or two types of NLSs, and vertebrate DNA2 proteins lack all three. Furthermore, mammalian DNA2 lacks the amino acids found in the N-terminal regulatory domain of *sc*DNA2. These findings suggest that, although eukaryotic DNA2 proteins have similar functions in DNA replication and repair, the mechanisms regulating them likely differ across organisms.

The participation of DNA2 in various DNA metabolic pathways is controlled by several factors. Many DNA replication and repair proteins, as well as post-translational modifications of DNA2, have been found to stimulate its ability to efficiently resect DNA ends, degrade regressing or regressed forks, or cleave DNA flaps. These activities are necessary to promote genome integrity in normal cells, as functional deficiency of DNA2 has been shown to cause genome instability and promote cancer initiation in mammals (21). Thus, DNA2 is considered to be a tumor suppressor. However, some DNA2 interaction partners are required to inhibit its activity to avoid the deleterious effects of its uncontrolled action on DNA intermediates. For instance, over-resection of stalled or regressed fork structures by DNA2 may cause fork collapse, increasing genome instability (22). Furthermore, DNA2 is overexpressed in several human cancers and has been found to support cancer cell survival by counteracting DNA replication stresses (23). Therefore, DNA2 may serve as a target for killing cancer cells or sensitizing cancer cells to existing chemotherapeutic agents.

## ENZYMATIC ACTIVITIES OF DNA2

Various biochemical characterizations of DNA2 proteins from different model organisms have shown that DNA2 possesses structure-specific nuclease, helicase and ATPase activities (7,10,15–17,19,20,24,25). It is now also commonly accepted that the DNA-dependent ATPase domain of DNA2, probably in coordination with its nuclease domain, functions as an ssDNA translocase in end resection during DSB repair (26,27). The enzyme activities of DNA2 require the presence of divalent cations, but the optimal concentrations required vary for each activity (e.g. 2.5–10.0 mM of Mg^2+^ for optimal nuclease activity, 1–2 mM for optimal helicase activity and 0.15–0.30 mM for optimal ATPase activity) (7,28). DNA2 also requires an intact Fe–S cluster to support both its nuclease and ATPase motor activities (13). In addition, its helicase and nuclease activities are modulated by ATP, as high (≥2 mM) concentrations of ATP reduce its nuclease activity, which likely causes the stimulation of its helicase activity (28,29). Indeed, robust helicase activity is only observed when the nuclease is attenuated by mutations or by high levels of ATP (7,30). Nevertheless, these observations do not rule out a role for the helicase in other processes mediated by DNA2 (31).

The DNA2 nuclease preferentially cleaves ssDNA from either a 5′ or 3′ end, but it can also cleave the ssDNA strand within a 5′ flap structure. The 5′ nuclease activity of DNA2 can be blocked by either an annealed oligonucleotide or a streptavidin–biotin conjugate at the 5′ terminus, demonstrating that, like flap endonuclease 1 (FEN1), DNA2 requires a free 5′ end (32). Unlike FEN1, however, DNA2 can also be blocked by a branch in the flap, indicating that it may also use a threading mechanism and track on ssDNA to reach the point of cleavage (32). The DNA2 helicase can unwind DNA duplexes to generate ssDNA regions (7,30), but unlike other known helicases, it requires the presence of a single-stranded 5′ end adjacent to the double-stranded DNA (dsDNA) to be unwound (33). Indeed, a detailed study of DNA2–DNA binding showed that DNA2 binds preferentially to the junction between ssDNA and duplex DNA. DNase1 footprinting revealed that DNA2 protects both the ssDNA, as well as the junction between the ssDNA and dsDNA (i.e. the base of the flap) (34).

**Figure 1.**
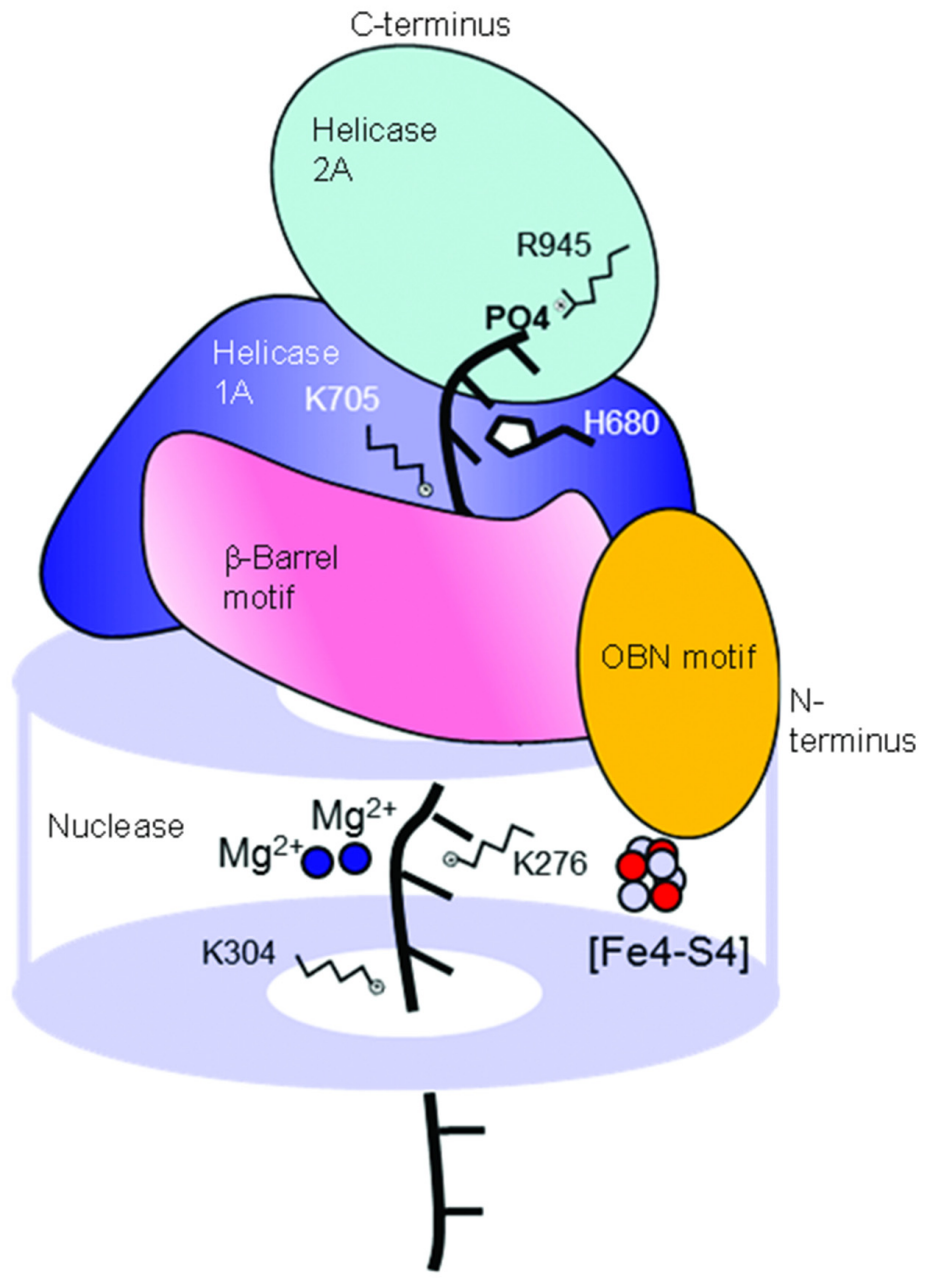
Schematic of the DNA2–DNA complex, elucidating its overall structure and the proposed mechanism for substrate binding and cleavage. Mouse DNA2 has an overall cylindrical shape. A narrow central tunnel is formed within the nuclease domain at the base and is extended by a β-barrel motif and a helicase 1A domain in the middle. A helicase 2A domain sits atop the tunnel. Several residues important for DNA substrate binding are indicated along the tunnel. The central tunnel is too narrow to allow dsDNA to access the active center in the nuclease domain. However, ssDNA can enter the tunnel through the bottom or top and thread to the other side. This schematic is based on previously published crystal structure information (35), with the two Ca^2+^ ions in the crystal structure replaced by two Mg^2+^ ions to reflect the role of Mg^2+^ as a co-factor for DNA2 catalysis.

Crystal structures of full-length mouse DNA2 in complex with a 15-nt ssDNA oligonucleotide illustrate, in part, how DNA2 recognizes and cleaves its DNA substrate (35). The DNA2-ssDNA complex displays an overall cylindrical shape with a long, narrow central tunnel (Figure 1). The nuclease domain is at the base of the cylinder; a β-barrel motif and stalk, as well as the helicase 1A domain, are on the top of the nuclease domain; and the helicase 2A domain is at the top of the cylinder. Because only ssDNA was in the structure, the binding site for dsDNA was not revealed; however, in keeping with the DNase I footprinting studies, it is predicted to be at the base of the nuclease domain (34). The nuclease active center and the DNA-binding motifs of the nuclease and helicase domains are enclosed in the central tunnel. The narrow tunnel allows only ssDNA to thread through, with the 5′ end of the ssDNA positioned at the DNA-binding motifs of the motor domain and the 3′ end positioned at the nuclease domain (35). These structural limitations explain why DNA2 preferentially acts on DNA substrates with free ssDNA ends and without branches or secondary structures (7,32,34).

## FUNCTIONAL ANALYSES of DNA2 BASED ON CELLULAR PATHWAYS

### Okazaki fragment maturation

The synthesis of Okazaki fragments on the lagging strand DNA template is initiated by the Pol α/primase complex, which synthesizes RNA–DNA primers. The primers consist of a RNA (7–14 nt) followed by a short stretch (10–20 nt) of DNA (36,37). The RNA-DNA primers are extended by Pol δ (DNA polymerase delta) in a series of discrete Okazaki fragments, which are about 200 nt in length (38–41). Through strand displacement, Pol δ creates RNA–DNA flaps that must be endonucleolytically removed (42,43) so that the Okazaki fragments can be joined into an intact lagging DNA strand. In the case that Pol α incorporation errors are introduced, the Pol α-synthesized DNA portion may be via the long flap cleavage or the FEN1-mediated error editing mechanisms (2).

Several different pathways contribute to flap removal and ligation. Most flaps are removed by FEN1 (2,41,44,45); however, the genetic and physical interactions between *sc*DNA2 and *sc*FEN1 (Rad27) suggested that DNA2 also plays a role in flap removal during Okazaki fragment maturation (46). This hypothesis was supported by the observation that DNA synthesized in a temperature-sensitive *dna2* mutant was shorter than full-length (7). Mutations in the *sc*DNA2 nuclease active site revealed that its nuclease activity is necessary for the essential functions of *sc*DNA2 in DNA replication (10,47). However, the helicase activity of DNA2, which is crucial for DNA replication *in vivo* (7,48), was thought to facilitate formation of short DNA flaps for removal (10,24,49). In addition, *sc*DNA2 helicase activity was shown to resolve secondary structures to facilitate the *sc*FEN1-mediated cleavage of RNA-DNA flaps (49). Nevertheless, *sc*DNA2 helicase activity was proven to be dispensable for yeast chromosomal DNA replication, though growth is inhibited in its absence and it is required for repair of methyl methanesulfonate-induced DNA damage (9,49,50).

**Figure 2.**
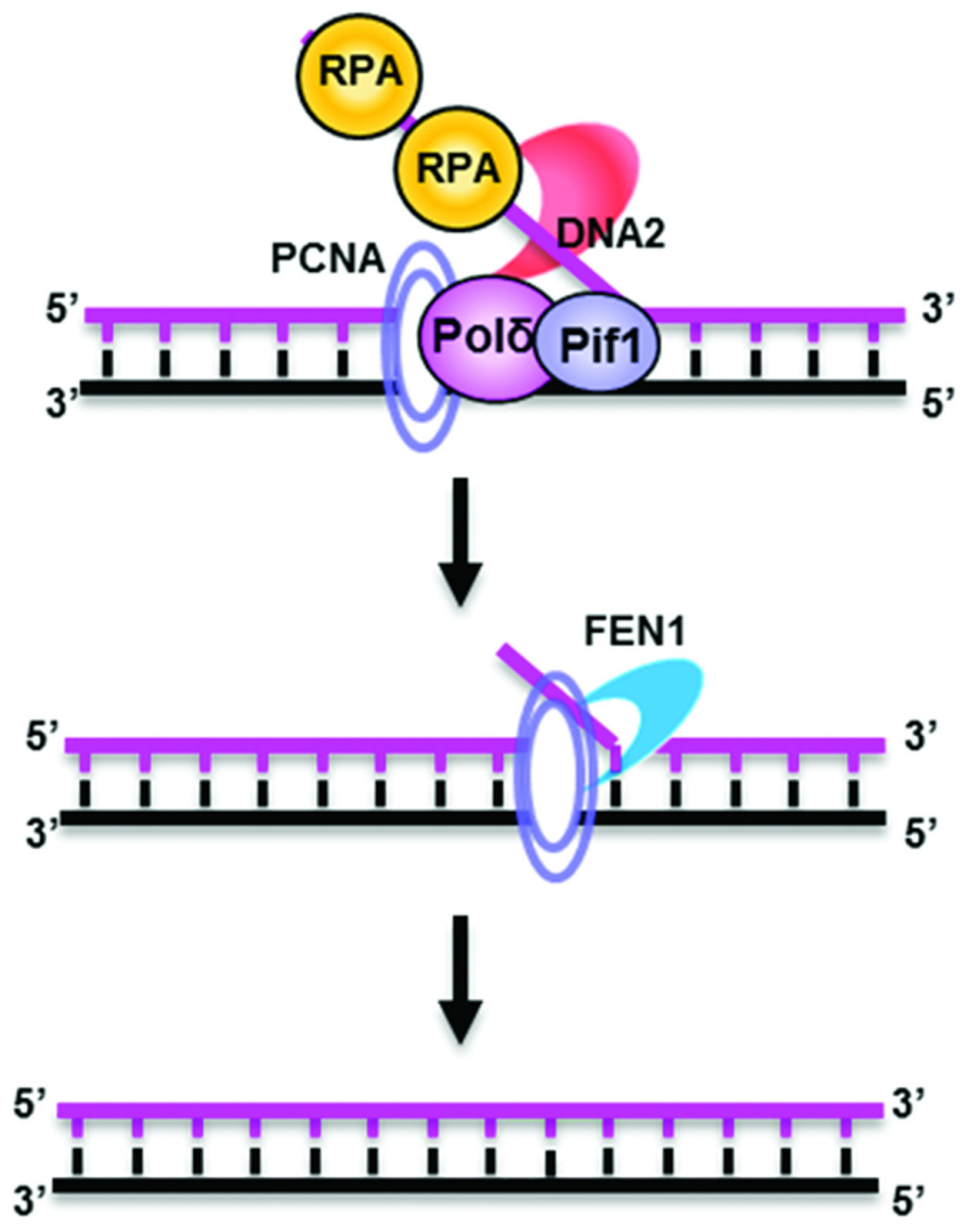
Sequential actions of DNA2 and FEN1 to remove a long RNA–DNA flap during Okazaki fragment maturation. RPA binds to long 5′ RNA-DNA flaps generated by Pol δ/PCNA and/or PIF1. The flap-bound RPA inhibits the action of FEN1 on the flaps and simultaneously recruits and stimulates DNA2 to cleave at the middle of the ssDNA strand, generating a shorter (∼8 nt) 5′ flap. FEN1 then dislodges DNA2 and cleaves at the junction between the ssDNA and dsDNA strands. DNA2 can also function alone to process long flaps. The RPA-mediated sequential actions of DNA2 and FEN1 or of DNA2 alone produce ligatable DNA nicks that can be joined to form intact lagging strand DNA.

These observations ultimately gave rise to a model that integrates multiple pathways of Okazaki fragment processing, each of which consists of 5′ flap structure removal before ligation. In one pathway, when an active Pol δ/PCNA complex encounters the RNA-DNA primer of the downstream Okazaki fragment, it displaces a short segment of a single-stranded RNA (ssRNA)-DNA flap of 2–10 nt (2), which is removed by FEN1 in a nick-translation reaction until the RNA segment is removed and a ligatable nick is available. If FEN1 is compromised, another structure-specific nuclease, exonuclease 1 (EXO1), can partially compensate by removing short flaps. In addition, it was proposed that Pol δ/PCNA can also give rise to long flaps and that both FEN1 and DNA2 could function in long flap removal. However, long flaps can be bound by replication protein-A complex (RPA), which inhibits FEN1 (11). Thus, in the presence of RPA, FEN1 alone can remove short flaps of less than 10 nt but not flaps longer than 30 nt (11,51,52). Interestingly, under conditions in which the *sc*DNA2 helicase was active, the presence of *sc*DNA2 stimulated scFEN1 to cleave longer flaps (11), even when they contained CTG fold-back secondary structures (52). This model suggested that FEN1 acts on most Okazaki fragments, but DNA2 is required for cleaving long flaps and flaps with secondary structures. In fact, the situation is even more complex. Further analysis revealed that DNA2 and FEN1 act sequentially in the long-flap pathway (Figure 2). First, DNA2 is recruited by RPA to static long-flap substrates. After it displaces the RPA, it tracks along and cleaves the flap to 5–7 nt, reducing its affinity for RPA. FEN1 then displaces DNA2, tracks along the remaining short flap, and cleaves at the base of the flap, creating a ligatable nick (11,53,54).

The physiological significance of the long-flap pathway remained unknown but was partially addressed by the identification of a new pathway component: PIF1 helicase. *sc*DNA2 was known to function at telomeres, as was PIF1 (50,55). During genetic studies of epistasis between DNA2 and other telomeric proteins, it was found that deletion of *PIF1* (*pif1Δ*) suppressed the lethality of nuclease-dead *dna2* mutants and even of complete *DNA2* gene deletion in *S. cerevisiae* (56). In *S. pombe*, a temperature-sensitive mutation of *pfh1* (which encodes Pif1) also suppressed the loss-of-function phenotype in a *dna2* mutant with temperature-sensitive mutations in the helicase domain (57,58). Thus, whereas the *sc*DNA2 nuclease is essential in normal yeast cells, it is dispensable in the absence of PIF1 helicase. To explain this phenomenon, it was proposed that PIF1 associates with Pol δ to processively displace RNA–DNA flaps, which require the long-flap pathway for processing (Figure 2). Indeed, using a substrate that contained a circular DNA template with an upstream oligonucleotide primer separated by a gap from a downstream oligonucleotide, it was demonstrated that Pif1 and Pol δ/PCNA generated long flaps that required both DNA2 and FEN1 to produce ligatable nicks (42,59,60). In addition, it had previously been shown that scFEN1 inefficiently cleaves short flaps (61), and even in the absence of long flaps, the long-flap pathway proteins (PIF1, RPA and *sc*DNA2) were shown to stimulate the FEN1-mediated short-flap pathway (62). Thus, the long-flap pathway proteins contribute to the flexibility of Okazaki fragment processing in at least two ways: (i) processing long flaps and (ii) stimulating the short-flap pathway. In addition, several studies have also suggested the existence of a long-flap pathway in which DNA2 functions alone, without FEN1 (63,64).

The potential toxicity of long flaps raises a fundamental question: What accounts for the evolution and retention of long flap production and processing? One interesting proposal is that DNA2, in order to facilitate the replication of difficult templates, has ‘hi-jacked’ the break-induced replication (BIR) process, in which PIF1 facilitates strand displacement by Pol δ to produce migrating bubble structures through which DNA synthesis can proceed (65–67). However, Okazaki fragment processing is more important than BIR for cell viability, so it is more likely that that PIF1 evolved to play an essential role in generating ligatable Okazaki fragments. This model posits that the hundreds of thousands of Okazaki fragments in yeast, as well as the millions in metazoans, render inevitable the stochastic failure of FEN1, necessitating the existence of efficient backup pathways. EXO1 provides a backup for short flap processing, but *S. cerevisiae exo1 rad27* double mutants are viable, suggesting that an additional pathway exists. This essential failsafe is DNA2. However, DNA2 cannot act on short flaps. Thus, the role of PIF1 is likely to provide an opportunity for DNA2 to participate in Okazaki fragment processing by creating long flaps that can recruit DNA2 for efficient cleavage. In the absence of PIF1, DNA2 cannot backup FEN1. In keeping with this, whereas *pif1Δ* suppresses the lethality of *DNA2* loss, it causes synthetic sickness with *rad27Δ* and synthetic lethality with *rad27Δ exo1Δ* (68). In addition, *pif1Δ dna2Δ* is synthetically lethal with deletions of genes encoding RNAse H2 subunits, *rnh202Δ* and *rnh35Δ*, suggesting that an RNAse H/FEN1 pathway may come into play when DNA2 cannot be recruited to long flaps (44,69). Finally, *pif1Δ* may not be lethal due to backup provided by helicase Rrm3. In *S. pombe*, which lacks RRM3, PIF1 is essential. In Okazaki fragment processing, therefore, there are many ways to ‘skin the cat.’

The PIF1 studies provided the first formal, albeit indirect, evidence for the accumulation of long-flap Okazaki fragment intermediates *in vivo* in the absence of *sc*DNA2. When *PIF1* was intact in *dna2* mutants, the S phase checkpoint, which can be triggered by the ssDNA-binding protein RPA, was strongly induced; however, deletion of *PIF1* or *POL32*, which encodes the processivity subunit of Pol δ, prevented checkpoint activation (56,65). This suggested that, upon loss of *sc*DNA2 alone, long flaps accumulated in numbers sufficient to bind enough RPA to induce the DNA replication checkpoint. Interestingly, deletion of the checkpoint mediators *RAD9* and *MRC1* also suppressed lethality in *dna2* mutant yeast, suggesting that the *sc*DNA2-deficient cells were killed due to checkpoint induction by immature Okazaki fragments (8,9,65,70). Recently, using electron microscopy, long flaps (median of ∼100–150 nt, but some longer than 1000 nt) were directly shown to accumulate behind replication forks *in vivo* in *S. pombe dna2* or *rad2* (Fen1) mutants (71). Electron microscopy also directly demonstrated that long flaps accumulated in *S. cerevisiae dna2* mutants and that such accumulation was reduced by 90% when Pif1 was ablated using a *pif1-m2* mutation in a *dna2Δ* strain (65). Finally, conventional *in vivo* pulse-labeling experiments and gel electrophoresis of nascent DNA in yeast showed that Okazaki-sized fragments accumulate at telomeres, but not at ribosomal DNA (rDNA), in the absence of either *sc*DNA2 or *sc*FEN1 (72). Taken together, these results strongly support that the function of *sc*DNA2 in the removal of long RNA–DNA flaps is essential for DNA replication and cell viability in yeast.

However, deep sequencing of Okazaki fragments in yeast was recently used to test the relative contributions of the various Okazaki fragment processing pathways *in vivo*. Surprisingly, *sc*DNA2 depletion did not significantly affect the maturation of the sequences queried by this technique (45). This result remains puzzling, considering the abundance of evidence suggesting, as summarized above, that the long-flap pathway is also important *in vivo*. The explanation perhaps lies in the details of the Okazaki fragment sequencing technique or in the fact that engagement of the long-flap pathway, though essential, is statistically very rare. In addition, experiments in human cells revealed that yet another pathway, mediated by human poly (ADP-ribose) polymerase (PARP) and the SSB repair protein XRCC1, might also be involved in the maturation of unligated Okazaki fragments that appear as SSBs, gaps, or flaps, and might be induced in the absence of DNA2 (73).

Indeed, in human cell lines, replication forks do not proceed more slowly after knockdown of *DNA2*, as shown by fiber tracking (74). Furthermore, homozygous *Dna2* knockout mice are viable until embryonic day 8.5 (E8.5), and *Dna2* knockout in mouse embryonic stem cells only slows DNA replication and is not lethal (21,75). These results suggest that DNA2-mediated RNA-DNA primer removal is not an essential pathway for Okazaki fragment maturation in mammalian cells, perhaps due to the PARP pathway and greater flexibility among the various Okazaki fragment-processing enzymes. Nevertheless, human HCT116 cells also lose viability precipitously after *DNA2* ‘shut-off’ [(76) and unpublished], suggesting an essential function of DNA2 other than Okazaki fragment maturation during DNA replication and cell proliferation.

### Facilitating the replication of ‘difficult-to-replicate’ DNA regions

**Figure 3.**
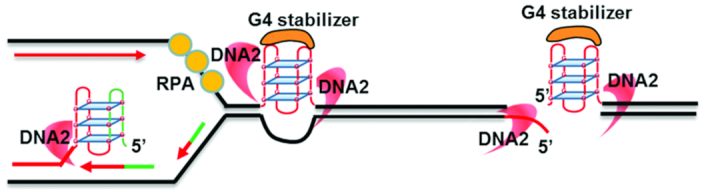
Roles of DNA2 in resolving DNA secondary structures, as typified by G4s, to facilitate DNA replication and repair. DNA2 endonuclease activity can directly excise G4s obstructing DNA replication fork movement. The resulting ssDNA gap is repaired by high-fidelity SSB repair. DNA2 can also remove G4s from DSBs to enable efficient end resection. The resolution of G4s by DNA2 is particularly crucial in the presence of G4 stabilizers that inhibit G4 unwinding by other helicases.

Across the genome, there are many fragile, or ‘difficult-to-replicate,’ sites that pose challenges for the replisome and act as an endogenous source of replication stress. Cells have developed several mechanisms to facilitate the replication of these sequences. DNA2 in particular has been shown to be a key enzyme in facilitating the replication of the major difficult-to-replicate regions, rDNA, telomeres and centromeres, which contain various types of putative replication fork-blocking structures (Figure 3) (21,55,77,78).

rDNA loci, which encode the ribosomal RNA genes, are organized in clusters of tandem repeats. It is estimated that there are 100–150 repeated ∼9–10-kb rDNA units in the rDNA loci of *S. cerevisiae* (79). Also within rDNA loci are *cis*-elements (∼100 bp), namely replication fork barriers (RFBs), that are tightly bound by the RFB-binding protein *sc*Fob1 (80). These RFB-Fob1 complexes must be resolved or bypassed during DNA replication. Yeast genetic studies revealed that helicases, including *scDNA2*, RecQ-like *SGS1*, and *RRM3*, are required to enable replication forks to move through RFBs in rDNA regions (78,81–83). Genetic deficiencies in *scDNA2*, *SGS1* or *RRM3* cause replication fork pausing, and deficiencies in *scDNA2* and *SGS1* additionally lead to DSB formation in the rDNA region. In contrast, Pif1 helicase helps maintain RFBs, which were less common in *pif1* mutants (83). The rDNA pause phenotype in *dna2*, *sgs1* and *rrm3* mutant strains was suppressed upon deletion of the *FOB1* gene, suggesting an important role of these helicases in resolving the RFB–Fob1 complex to facilitate DNA replication at rDNA loci. These helicases may directly promote replication fork progression through the protein-DNA complex and/or participate in the resolution of converged DNA replication forks formed at the RFBs. In keeping with this idea, *sc*DNA2 was also found to interact with genes encoding Pol α and CTF4/AND-1, the latter of which is now known as a hub for the interaction of the CMG (Cdc45-MCM-GINS) helicase complex with Pol α and other proteins, including DNA2 (9,84). The DNA2 and CTF4/AND1 interaction has been proposed to be significant for rDNA maintenance in both yeast and human (78,82,84).

Telomeres are specialized DNA-protein structures that protect chromosome ends from inappropriate degradation and fusion (85,86). They are greatly important for maintaining genome integrity and avoiding neoplastic transformation. Telomeres not only cap chromosome ends but also have a reversible ‘telomeric silencing’ effect on genes internal to the terminal repeats. However, overexpression of *scDNA2* disrupted the silencing of genes inserted into yeast subtelomeric DNA (87). Study of this phenomenon showed that *sc*DNA2 localizes to telomeres in a Sir3-dependent and cell cycle phase-specific program (55). It localizes to telomeres during the G2 phase and is also present at telomeres in G1. In S phase, however, it relocates to origins of replication. *sc*DNA2 also delocalizes from telomeres when DSBs are introduced upon bleomycin treatment. DNA2 was also shown to localize to telomeres in mammalian cells via interactions with telomere-binding factors TRF1 and TRF2 (21). Based on the interaction of scDNA2 with Sir3 at telomeres, as well as its role in rRNA stability, its contribution to aging was investigated in yeast. *dna2* mutants had shortened replicative life spans, indicative of premature aging (88). In humans, a splice-site mutation that leads to decreased levels of DNA2 was found to enhance cellular senescence and gives rise to Seckel syndrome, a type of primordial dwarfism (PD) (89), thus illustrating the conserved impact of DNA2 on aging and development.

At least one function of DNA2 at telomeres may involve secondary G-quadruplex (G4) structures, which form in regions of guanine (G)-rich ssDNA (85). Indeed, telomeres consist of long tracts of G-rich tandem repeats [(TTAGGG)n in humans], and G4 structures have been detected at telomeres using structure-specific antibodies and proteins that recognize G4s in cells (90–93). G4s are defined by stacks of two or more G-quartets, which are formed by four Gs associated via Hoogsteen base-pairing, stabilized by a monovalent cation (94). G4s are particularly problematic for DNA replication machinery, because they form spontaneously and are thermodynamically stable (95,96). Unresolved G4s can block replication fork progression, and ligands that stabilize G4s inhibit telomere DNA replication, suggesting that G4 DNA is pathological (95). Many pathways have been found to resolve and/or clean up G4 barriers to allow efficient DNA replication. The most well-known mechanism is G4 unwinding by DNA helicases, including FANCJ, PIF1, RTEL1, RECQ5, BLM, WRN and G4R1 (97–104). These helicases have been shown to promote DNA replication at telomeres and other G4-forming sequences. In addition, the DNA replication protein complexes RPA (RPA1, RPA2 and RPA3) and CST (CTC1, STN1, and TEN1) can directly unfold G4 structures (105,106). However, the unfolding or unwinding of G4s typically requires an ssDNA tail on which to load the RPA, CST or G4 helicases (102,105,106). Moreover, G4-stabilizing chemicals and G4-binding proteins significantly inhibit the ability of helicases to resolve G4s (102). To ensure protection against a potential lack of an ssDNA tail and/or G4 stabilization, another mechanism is needed to resolve G4s. Both *sc*DNA2 and mammalian DNA2 can recognize and effectively unwind and cleave G4 structures, presumably removing the G4s ahead of the replication fork to facilitate fork progression (Figure 3) (21,107). In fact, *sc*DNA2 deficiency results not only in telomere shortening but failure to join telomeric Okazaki fragments (55,72), suggesting roles in the resection and replication of telomeres. DNA2 deficiency in mammalian cells also leads to telomere fragility and shortening, as well as hyper-recombination between sister chromatids (21). These results suggest a conserved role of DNA2-mediated G4 resolution in facilitating telomere DNA replication and perhaps replication of G-rich chromosomal sequences.

Centromeres, which orchestrate chromosome segregation, contain the largest clusters of tandem repeats in the human genome, namely α satellite DNA (108). The basic units of α satellite DNA consist of 171-bp sequences, which form highly homologous arrays of up to several million base pairs at the centromeres of all human chromosomes. These α satellite repeat sequences also place a burden on DNA replication machinery, due to their tendency to form various secondary structures on both the DNA template and the ssDNA flaps of the newly synthesized daughter strand DNA (109). Factors that facilitate centromere DNA replication were unknown until recently. A study using proteomic and biochemical approaches demonstrated that the DNA repair proteins MSH2 and MSH6 are crucial for efficient centromere DNA replication (110). Importantly, this study also demonstrated that inhibition of RPA and the downstream kinase ATR is critical for centromere DNA replication (110). Chromatin immunoprecipitation sequencing technology revealed that, under normal physiological conditions, nuclear human DNA2 localizes predominantly to centromeres (77), and single molecule analysis of replication dynamics (SMARD) and other techniques revealed that DNA2 has several functions during centromere DNA replication (77). First, its helicase activity can help to resolve stem–loop structures on the DNA template strand (Table 1). Second, its helicase and nuclease activities can work together to effectively remove RNA-DNA flaps that contain secondary structures (Table 1). Third, it may help to process the centromere DNA to ensure the proper formation of secondary structures that have recently been found to be critical for loading the centromere-binding protein CENPA and for suppressing RPA binding and ATR activation at centromeres. Supporting this postulated function, DNA2 deficiency was found to impair CENPA loading but enhance RPA binding and ATR activation at centromeres (77).

### End resection for checkpoint activation, homology-directed repair and telomere end protection

DNA molecules in eukaryotic cells constantly experience DNA damage. DSBs, due to endogenous or environmental genotoxic insults, constitute the most lethal and mutagenic type of DNA damage (111–113), and cells must repair them immediately and with high fidelity. Unrepaired DSBs can cause apoptosis or cellular senescence, whereas improper repair can cause chromosomal translocations and deletions (111–113). As a result, highly sophisticated and conserved systems have evolved in eukaryotic cells to rapidly detect and efficiently repair DSBs. To date, three major pathways for DSB repair have been defined: homology-directed repair (HDR), classical non-homologous end-joining (c-NHEJ), and alternative non-homologous end-joining (Alt-NHEJ) (114). HDR requires extensive DSB end resection to generate 3′ ssDNA overhangs that subsequently invade homologous DNA duplexes to create D-loop structures. These homologous recombination intermediates are unwound, resulting in high fidelity repair of DSBs. The 3′ ssDNA overhangs also serve as signals to induce activation of ATR signaling. In contrast, c-NHEJ, which is also highly accurate, requires no DNA end resection. Instead, c-NHEJ machinery directly joins broken DNA ends. The third pathway, Alt-NHEJ, involves limited DNA end resection to generate regions of micro-homology to facilitate annealing and end-joining. This error-prone pathway frequently causes chromosomal translocations.

HDR is initiated by DNA end resection of DSBs (113). The 3′ overhang of the resected ends recruits RPA and activates the DNA replication or DNA damage checkpoint through CHK1 or CHK2, depending on the nature of the damage (115). RPA is then replaced by Rad51, and the Rad51 filament initiates HDR. The observation that yeast *dna2* mutants were sensitive to X-rays provided the first indication that DNA2 may participate in DSB repair (116). In addition, its nuclease/helicase activities were strikingly similar to those of RecBCD, which is the primary end processing enzyme in *E. coli* (10). A role in DSB repair was also suggested by a global synthetic lethal screen, which unexpectedly revealed that *dna2* mutations were synthetically lethal with mutations to the DNA repair protein Sgs1 but not with mutations to DNA repair proteins Rad51, Rad55, Rad57 and Rad59 (69). In addition, BLM, the human ortholog of Sgs1, was also shown to suppress the DNA repair defects of yeast *dna2* mutants and to interact with *sc*DNA2 (117). This is consistent with subsequent genetic and biochemical studies, which indeed defined DNA2 as a critical nuclease that acts in conjunction with RecQ helicases (Sgs1/BLM) to resect 5′ ends of DSBs for checkpoint activation and HDR in yeast (118–122). The function of DNA2 in DSB end resection is also conserved in higher eukaryotic organisms, including *Xenopus* (123,124), chicken (125), and human, as shown by the accumulation of RPA and Rad51 foci upon DNA2 depletion and through other biochemical methods (126,127). In *S. cerevisiae*, the MRX (Mre11-Rad50-Xrs2) complex or Sae2 nuclease (CtIP in humans) initiates 5′ degradation of broken ends, and an Sgs1/*sc*DNA2 complex continues to cleave the DNA in the 5′-to-3′ direction to generate an extensive 3′ ssDNA overhang (118,121,122).

Using point mutations that specifically eliminate the helicase activity of Sgs1 or *sc*DNA2, it was found that the Sgs1 helicase, but not the *sc*DNA2 helicase, unwinds the DNA duplex to create ssDNA flaps (121). In single-molecule studies, it has been shown that Sgs1 is recruited to DNA ends through either Top3-Rmi1-dependent or -independent pathways (128). These studies also showed that the Sgs1 end processing machinery can rapidly displace *in vitro* reconstituted nucleosomes, but is not activated until *sc*DNA2, including nuclease- and ATPase-dead *sc*DNA2, is added. Once the ssDNA overhangs have been generated, the *sc*DNA2 nuclease is activated for resection, but only in the presence of RPA. RPA binds and sequesters the ssDNA unwound by Sgs1. Single-molecule analysis of human DNA resection proteins identified phospho-RPA as an inhibitor of the BLM helicase during DNA2- or EXO1-mediated resection (129). Importantly, the binding of RPA to ssDNA stimulates the 5′ nuclease activity and inhibits the 3′ nuclease activity of *sc*DNA2, thereby leading to selective 5′ end resection by scDNA2 (107,118,121). The crystal structure of a peptide containing the α1 helix of human DNA2 (residues 1–20) in complex with the OBN domain of human RPA70 (residues 1–120) explains how RPA regulates the polarity of DNA2 nuclease activity (35). In yeast cells, Exo1, redundantly with the Sgs1/scDNA2 complex, mediates long-range end resection (122). *Sc*DNA2- and Exo1-mediated end resection is both positively and negatively regulated by the 9-1-1 checkpoint clamp and negatively regulated by the checkpoint mediator Rad9 (53BP1 in humans) (130). However, the yeast chromatin remodeler Fun30 (SMARCAD1 in humans), which is required for resection *in vivo*, is thought to overcome inhibition by Rad9 (131–133). In *Xenopus*, the ortholog of helicase WRN (Ffa-1), another RecQ helicase, associates with Dna2 for resection (134).

Telomeres represent specialized DSBs. Immediately following telomere replication, the C-rich strands at the telomere ends are resected in a series of steps to generate G-overhangs, which form t-loop structures that protect the chromosome ends from being recognized as toxic, recombinogenic DSBs (135–137). Genetic studies in yeast suggest that *sc*DNA2 plays a critical role in telomere end resection to generate 3′ G-overhangs on the newly synthesized lagging strand (72,138,139). Another recent study, however, reported that the Apollo and EXO1 nucleases resect the nascent leading strand of mammalian telomere ends (135). These findings indicate that eukaryotic cells from different organisms use distinct resection mechanisms to process telomere ends for t-loop formation.

### Processing of stalled replication forks

Efficient and accurate DNA replication is essential to ensure that daughter cells faithfully receive genetic material and ultimately survive (140). However, DNA replication machinery is frequently challenged by DNA lesions, stable secondary DNA structures and DNA-bound protein complexes. In each of these situations, the replication fork stalls, and if repair is not efficient, the forks collapse into DSBs or other structures, notably gaps. The proper maintenance, processing, and restarting of the stalled or collapsed or uncoupled replication forks are of paramount significance for cells, as mishandling of the damaged forks can lead to genome instability, tumorigenesis, or cellular lethality (140). Thus, several mechanisms have evolved in eukaryotic cells to manage stresses on the replication fork. When replication forks transiently stall in response to replication stresses, electron microscopy reveals two major intermediates: single-stranded gaps either at or behind the fork and reversed fork structures (141). ssDNA is generated, in some cases due to the uncoupling of DNA polymerase from the replicative helicases, and/or by the processing of reversed forks (4,142). The accumulation of ssDNA can lead to the binding of RPA, which subsequently recruits and activates MEC1 in yeast cells or ATR in mammalian cells. Activated MEC1/ATR coordinates different pathways to stabilize, process, or restart the stalled replication forks, depending on the nature of the impediment, the structure of the forks, and the availability of DNA replication and repair proteins.

**Figure 4.**
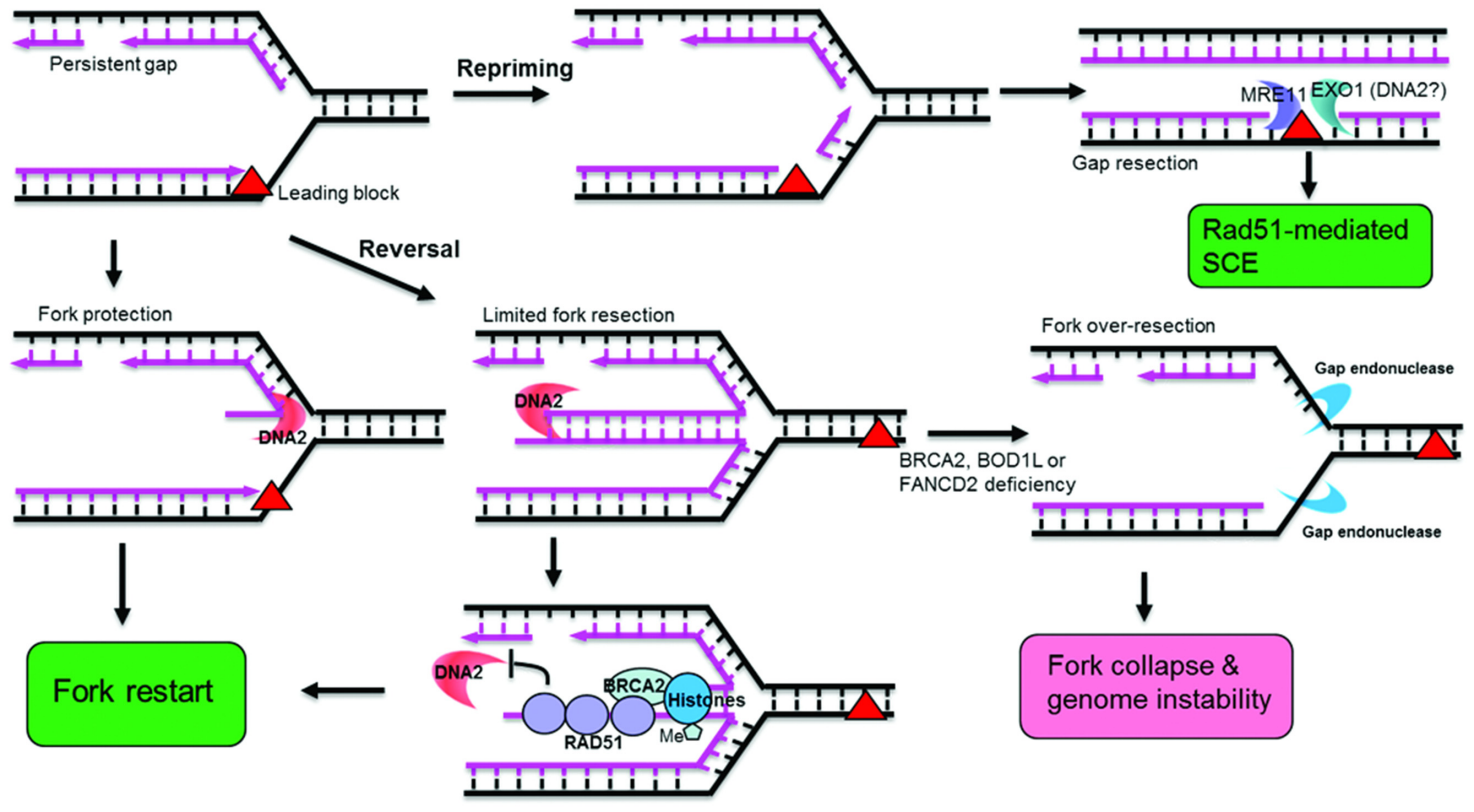
Multiple functions of DNA2 in stalled replication fork processing. DNA2 can participate in stalled replication fork protection, limited resection, or over-resection, depending on the nature of the fork and the availability of other fork-protecting factors. To protect a replication fork, the 5′ nuclease activity of DNA2 may cleave the 5′ ssDNA flap, preventing the reversal of nascent DNA, which can lead to the formation of potentially deleterious regressed fork structures. If regressed forks do arise, however, the 5′ nuclease activity of DNA2 may conduct limited 5′ resection, generating a 3′ ssDNA overhang to facilitate the helicase-driven regression of the regressed fork structure to restart the fork. Meanwhile, BRCA2 may be recruited to the regressed fork structure to limit the action of DNA2 to avoid over-resection. In addition, SET1A, in complex with BOD1L, catalyzes H3K4 methylation, which facilitates FANCD2-mediated histone assembly on the regressed fork and stabilizes the RAD51 filament on the nascent DNA to protect the fork before restarting. In the absence of BRCA2 or BOD1L, extensive degradation of the regressed fork by DNA2 and other nucleases may occur, leading to fork collapse and genome instability. In addition, the lesion that blocks leading strand DNA synthesis may be bypassed by the repriming process. The resulting gap will be processed and repaired post replication. The role of DNA2 in processing of the gap is undefined.

DNA2 has been defined as a central factor in counteracting stresses on replication forks. Its helicase and nuclease activities can resolve stable secondary structures, including stem–loops and G4s (Figure 3), thus removing intrinsic replication barriers and preventing replication fork stalling (21,77,107). Furthermore, when restarting stalled replication forks, it is crucial to maintain the proper configuration of the fork to avoid the formation of intermediate structures that will potentially generate deleterious gaps or DSBs. One such intermediate is the regressed fork structure, in which the template rewinds and the nascent leading and lagging DNA strands anneal to each other to form a four-way DNA structure resembling a Holliday junction (HJ). Although this is an important structure for restarting the fork, if repair is not efficient, it may be cleaved by HJ resolvases or endonucleases, such as Mus81, to create one-ended DSBs. These DSBs can be used for recombination restart but can also be toxic. DNA2 plays several roles in resolving regressed forks and in several other stages of stalled fork processing and restarting. For example, in *S. pombe*, in response to fork stalling induced by hydroxyurea, ATR is activated by phosphorylation. The active form of ATR can then phosphorylate Cds1 (a CHK2 homolog), which subsequently phosphorylates Dna2 at residue S220, which promotes the association between Dna2 and chromatin (143). Biochemical studies using model substrates that mimic four-way replication forks with nascent, unannealed 3′ and 5′ leading and lagging DNA strands showed that Dna2 is capable of degrading 5′ or 3′ ssDNA before the fourth arm can form (Figure 4) (143). In this way, DNA2 may help prevent deleterious consequences of fork reversal.

DNA2 can also directly act on regressed fork structures to facilitate their proper resolution and fork restart (Figure 4). The Vindigni group showed that limited DNA2 resection is required to restart forks arrested by hydroxyurea in human osteosarcoma U2OS cells (22). The DNA2-mediated degradation of nascent DNA at stalled forks is stimulated by WRN and RAD51 (144,145) but inhibited by RECQ1 (22). Specifically, DNA2 and WRN were shown to resect four-way DNA structures with a 5′-to-3′ ssDNA overhang on one arm *in vitro* (22), and RAD51 and RECQ1 were proposed, respectively, to increase and counteract fork reversal. Evidence for DNA2 degradation of DNA after fork reversal was provided by electron microscopy, which showed that DNA2 depletion resulted in significant accumulation of revered forks, even in unperturbed U2OS cells. Furthermore, more reversed forks were double-stranded in DNA2-depleted cells than in normal cells, suggesting that nascent DNA is degraded by DNA2 on the reversed fork structures, generating the ssDNA overhangs. Once forks are reversed, a motor protein, such as RecQ helicase or the DNA translocase SMARCAL1, can act via a branch migration mechanism to restart the partially resected, reversed forks with 3′ overhangs (22,146). In yeast, *sc*DNA2 has also been implicated in reversed fork resolution because it stimulates EXO1 to resect the nascent, annealed regressed fork DNA to eliminate aberrant replication intermediates in cells that are replication checkpoint-defective (147). In addition, knockdown of Merit40, which is a fork protection protein involved in recruitment of BRCA1, also appears to synergize with DNA2 knockdown, suggesting that they participate in parallel processes of fork protection/restart (148). This pathway might occur at gaps, which may arise after nuclease degradation of DNA flanking interstand crosslink or DNA gaps being repaired by post-replication repair processes (6,149). It offers an alternative to reversed forks for replication restart (Figure 4). MRE11 or EXO1 is suggested to process the gap to initiate Rad51-mediated sister-chromatin exchange (SCE) (149). However, it is not known if DNA2 plays any role on these gaps.

DNA2 and other nucleases are critical for properly restarting stalled replication forks and for maintaining genome integrity; however, the uncontrolled activities of DNA2, EXO1 and MRE11 can also result in unscheduled or over-resection of stalled replication forks, contributing to genome instability (Figure 4) (141,150,151). Many factors have been found to regulate DNA2-mediated resection activity at stalled replication forks. Binding of the histone H3K4 methylase SETD1A or BOD1L, a SETD1A subunit, to the stalled replication fork stabilizes RAD51 and inhibits DNA2- and BLM-dependent fork resection (75,152,153). Interestingly, while RAD51 promotes the action of DNA2 on regressed forks during fork restart, probably by aiding in fork reversal, it also plays a critical role in protecting the fork from excessive degradation by DNA2 (56,69) and other nucleases (150,152), possibly by binding to the 3′ ssDNA overhang. DNA2-dependent fork resection is also controlled by ABRO1 (154), which is a paralog of the BRCA1-binding protein Abraxas (155). A more recent study suggested that CtIP is also critical in preventing DNA2-mediated over-resection of the stalled replication fork. It was postulated that CtIP, which inhibits EXO1 nuclease activity *in vitro* (156), controls the nuclease activities of DNA2 and EXO1 to limit resection. In addition, human RIF1 inhibits the phosphorylation of WRN and of DNA2 to limit the formation of DNA2-WRN complexes and inhibit fork degradation by DNA2 (157,158) . Finally, telomerase was also found to stabilize reversed replication forks that formed in telomeric DNA due to unresolved t-loops and G4s in RTEL1-deficient cells, potentially by preventing normal fork restart mediated by telomeric DNA2 (159).

New studies on epigenetic modifications and histone mobility suggest that events at stalled forks explain the old observation in yeast that *dna2-1*, *dna2-2* and *dna2Δ pif1Δ* mutations are synthetically lethal with mutations in chromatin remodeling and histone chaperone genes. Early on, it was found that *dna2-2* caused synthetic sickness with mutations to *spt16* and *pob*3, which are components of the FACT chromatin remodeling complex that interact with Pol α (69,160). It was proposed that these interactions might facilitate the progression of Pol α and DNA2 through nucleosomes. More relevant to replication fork stalling, it was demonstrated that *dna2* mutations were synthetically lethal with mutations to the Rad6/Bre1 complex, which is involved in histone H2B ubiquitination and is required for histone H3 lysine methylation, as well as with mutations to several genes in the COMPASS/Set1 histone methylase complex. This suggested a link between epigenetic modifications, DNA2 function, and genome stability, because the COMPASS mutants were hydroxyurea-sensitive.

The mechanisms underlying these synthetic lethality phenotypes are just beginning to emerge in human cells. Stewart group discovered that loss of human BOD1L, the SETD1A histone H3K4 methylase subunit, led to sensitivity to inter-strand cross-links due to loss of replication fork protection and over-resection of nascent DNA (153). They further suggested that H3K4 methylation by SETD1A promotes RAD51 filament formation, which limits DNA end resection by DNA2. They also showed that, in the absence of methylation, depletion of DNA2 suppressed inter-strand cross-link sensitivity and reduced resection of nascent DNA. Interestingly, the histone chaperone/nucleosome assembly function of FANCD2, a known fork-protection and inter-strand cross-link repair protein, is stimulated by H3K4, and suppression of either H3K4 methylation or the histone chaperone activity of FANCD2 led to over-resection that correlated with loss of RAD51 filament formation (Figure 4) (152,153). More recently, the Moldovan group showed that incomplete Okazaki fragment maturation and gap-filling due to defective PCNA ubiquination interferes with CAF1-driven histone deposition. They propose that the altered histone deposition process impairs protection of stalled replication forks, leading to DNA2-driven fork degradation (151). Clearly, the diverse, multi-layered reactions at stalled forks need to be reconstituted and studied biochemically, as was done to understand the sequential reactions during Okazaki fragment maturation, to test the many possible mechanisms and determine the specific contributions of each of these mechanisms and how they are used at different types of stalled forks.

### Mitochondrial DNA replication and repair

An unexpected function for DNA2 in mitochondria was discovered through studies instigating how inhibition of the nuclear functions of PIF1 suppresses the lethality of *dna2Δ* in yeast. The mutant *pif1-m2*, which has an intact mitochondrial localization signal but an inactivated NLS, demonstrates proficient mitochondrial replication and can grow on non-fermentable carbon sources but is deficient in nuclear Pif1 functions (which are not essential for viability). *pif1-m2*, like *pif1Δ*, was found to suppress the lethality of *dna2Δ*. However, the *pif1-m2 dna2Δ* double mutant lost mitochondrial DNA (mtDNA) rapidly and could not grow on non-fermentable carbon sources, establishing that DNA2 was necessary for mtDNA stabilization (56).

**Figure 5.**
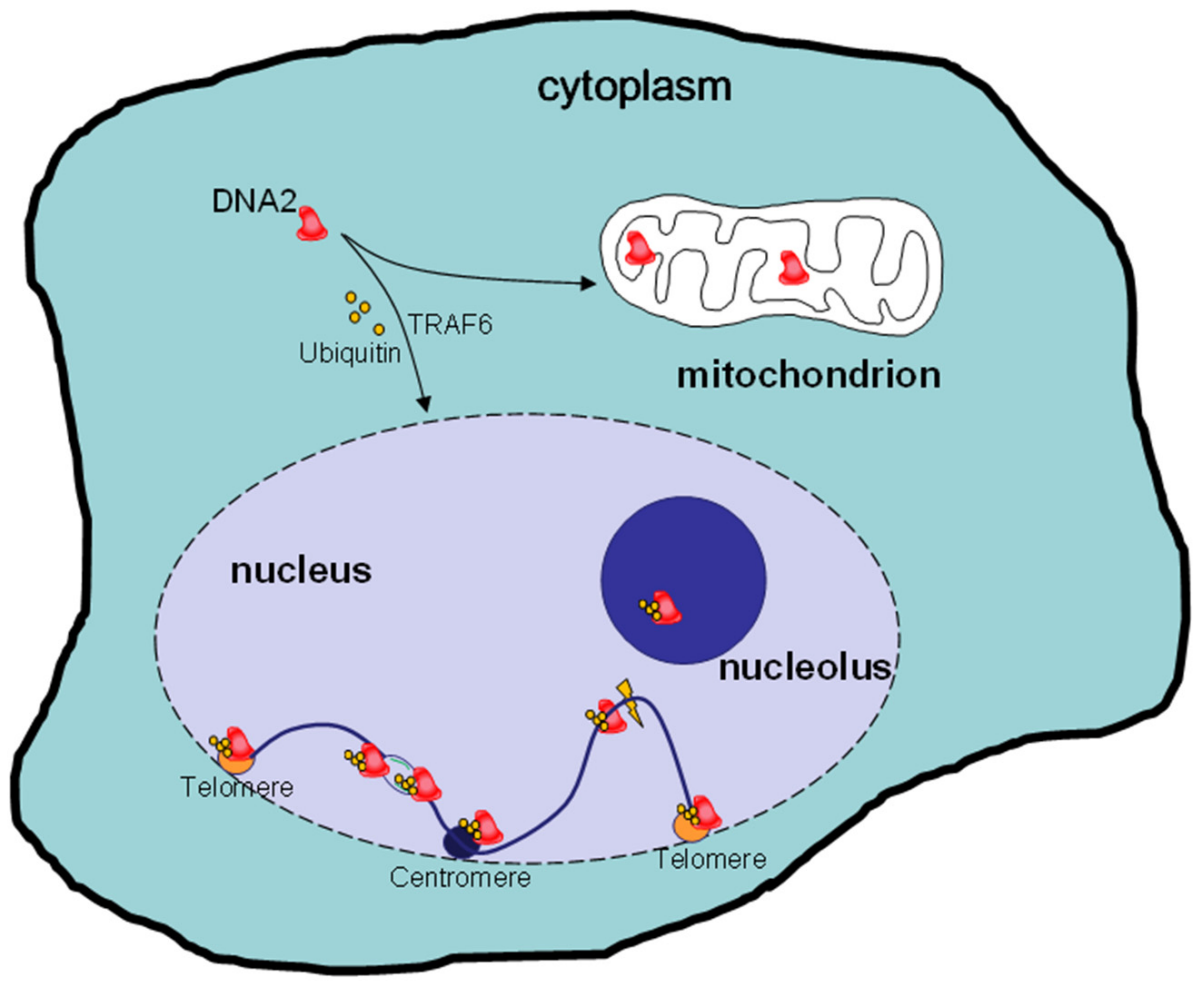
Mitochondrial localization and TRAF6-mediated nuclear translocation of human DNA2. Like its yeast counterpart, human DNA2 migrates into both mitochondria and nuclei. However, unlike *sc*DNA2, human DNA2 has no NLS and translocates into nuclei via an NLS-independent mechanism that depends on TRAF6-mediated polyubiquitination. Once in the nucleus, DNA2 can localize to telomeres, centromeres or the nucleolus to facilitate the DNA replication of these difficult-to-replicate regions. DNA2 can also be recruited stalled replication forks or DSBs to facilitate the repair of these intermediate structures via the HDR pathway.

The same was shown for metazoans. Mammalian mtDNA is a circular molecule of approximately 16 kb. Each cell contains hundreds to thousands of mitochondria, and each mitochondrion contains several copies of the mtDNA genome (161). mtDNA is typically replicated via a strand displacement mechanism (161), which requires unwinding of the DNA template by a helicase. Human DNA2 has been demonstrated to localize to mitochondria and to be required for the stability of mtDNA, as well as base excision repair (BER) (Figure 5) (14,162). Interestingly, human DNA2 has a predicted N-terminal mitochondrial localization signal peptide, but the peptide does not play a role in mediating DNA2 mitochondrial localization (14). Instead, a region within the helicase domain of DNA2 is essential for its mitochondrial localization. When this region is fused to GFP, the GFP localizes to mitochondria, suggesting that it is a new mitochondrial targeting motif (14). Mitochondrial DNA2 interacts with mitochondrial Pol γ and greatly enhances the activity and processivity of Pol γ-mediated strand displacement DNA synthesis *in vitro* (14). However, replication forks similar to those generated during nuclear DNA replication have also been observed in mtDNA using 2D gel electrophoresis, suggesting that replicating mtDNA also uses a strand-coupled DNA replication mechanism (163,164), which may involve the sequential actions of DNA2 and FEN1 to remove RNA–DNA flaps (14,162,165).

## REGULATORY MECHANISMS

### Protein-protein interactions

DNA2 forms complexes with various proteins, including DNA replication proteins, DNA repair proteins, telomere-binding proteins, and protein-modifying enzymes (Table 2). In general, these protein-protein interactions facilitate the functions of DNA2 in different DNA metabolic pathways by recruiting it to DNA replication or repair sites or by stimulating its nuclease or helicase activities. *sc*DNA2 interacts with RPA and FEN1 to efficiently process long flaps during Okazaki fragment maturation (11,12,14,46). RPA recruits *sc*DNA2 to long RNA-DNA flaps, stimulates its nuclease activity to cleave the long flaps, and coordinates its sequential actions with FEN1 (11). Additional studies revealed that RPA may stimulate the nuclease activity of *sc*DNA2 by melting the secondary structures on the 5′ flaps during Okazaki fragment maturation (54). Compared to *sc*DNA2, human DNA2 lacks approximately 400 amino acid residues in its N-terminus, including the RPA interaction motif (12,19,20). Thus, the effects of RPA on DNA2 likely differ between species. Indeed, Seo group suggests that RPA does not stimulate the human DNA2-catalyzed cleavage of 5′ ssDNA from the a duplex DNA substrates at low concentrations and inhibits such cleavage at high concentrations (19). However, studies from other groups suggest that the interactions between RPA, mammalian DNA2, and the DNA substrate ensure that DNA2 specifically cleaves the 5′ ssDNA strand and not the 3′ ssDNA strand during DSB end resection (127). In addition, RPA melts secondary structures on the 5′ ssDNA, further promoting the cleavage of 5′ flap by mammalian DNA2 (35). In fission yeast, it was shown that Dna2 interacts with the replication protein Cdc24 and that the Dna2/Cdc24 interaction may stimulate Dna2 to cleave the RNA–DNA flap (58). Intriguingly, no Cdc24 homologs have been identified in other species (58).

**Table 2.** DNA2 interaction proteins and the pathways in which the complexes are formed

Protein	Pathways	Organism	References
RPA	Okazaki fragment maturation	Sc. H.	(11,12)
RPA	DSB end resection	Sc. H	(118,127)
PCNA	Replication fork processing	H.	(23)
FEN1/Rad27	Okazaki fragment maturation	Sc. H	(14,46)
CTF4	Okazaki fragment maturation	Sc.	(9)
CTF4	DSB end resection	Sc.	(78,82,84,124)
Cdc24	Okazaki fragment maturation	Sp.	(58)
BRCA1	DSB end resection	C.	(125)
CtIP	DSB end resection	C.	(125)
Sgs1/WRN, BLM	DSB end resection	Sc. H.	(12,122,125,144)
9-1-1	DSB end resection	Sc.H.	(130)
FANCD2	Replication fork processing	H.	(126)
TRF1	Telomere replication	M. H.	(21)
TRF2	Telomere replication	M. H.	(21)
Pol γ	mtDNA replication, repair	H.	(14)
Cdk1	DSB end resection	Sc.	(167)
Cds1	Replication fork processing	Sp.	(174)
p300	UV damage repair	H.	(166)
TRAF6	Nuclear location	H.	(170)
MMS19	Iron-sulfur cluster assembly	Sc. H.	(168)

Sc.: *S. cereviasiae*, H.: human; M.: mouse; Sp.: *S. pombe*; C.: chicken.

In response to DNA replication stress, DNA2 interacts with PCNA and participates in processing and restarting stalled or collapsed replication forks (63). During DSB repair, DNA2 interacts with BRCA1, CtIP and RecQ helicases (Sgs1 in yeast or WRN and BLM in mammalian cells). BRCA1 and CtIP are crucial for recruiting DNA2 to DSB sites in human and chicken DT40 cells (125). The interaction between DNA2 and the RecQ helicases stimulates the nuclease activity of DNA2 (122,127,144). A recent study revealed that the DNA repair protein complex 9-1-1 also interacts with and facilitates the function of DNA2 in resecting DSB ends (130). In addition, DNA2 interacts with the inter-strand cross-link repair protein FANCD2 in U2OS cells, and knockdown of DNA2 suppressed the sensitivity of FANCD2-deficient cells to the DNA cross-linking agent cisplatin, suggesting a role for a putative DNA2/FANCD2 complex in the Fanconi anemia/BRCA pathway (126).

The interaction between DNA2 and the telomere-binding proteins TRF1 and TRF2 is important for the localization of DNA2 to telomeres and for DNA2-mediated end resection of telomere ends (21). In addition, mitochondrial DNA2 interacts with mitochondrial Pol γ for mtDNA replication and repair (14). Another category of DNA2 interaction partners are proteins that mediate the post-translational modification and co-factor assembly of DNA2, including p300 (166), Cdk1 (167), Cds1 (a CHK2 homolog) (143) and MMS19 (168), which catalyze the acetylation, phosphorylation, and Fe–S cluster assembly of DNA2, respectively.

### Post-translational modifications of DNA2

To date, three major post-translational modifications of DNA2 have been identified. Acetyltransferase p300 is reported to interact with and acetylate human DNA2 *in vitro* and in cells (166), and acetylation of DNA2 by p300 is stimulated by UV irradiation. Acetylated DNA2 has 10-fold higher DNA binding affinity, as well as greater nuclease, helicase, and ATPase activities, than non-modified DNA2 (166). Acetylation has been proposed to serve as a switch that directs RNA-DNA flap cleavage, because it inhibits FEN1 while stimulating DNA2, thus promoting the long-flap pathway (166). This switch might be useful in increasing the size of the patch of re-synthesis during Okazaki fragment processing or DNA repair, providing a greater opportunity for the correction of error-prone synthesis by Pol α or of damaged bases, respectively (166). Phosphorylation has been reported to play a crucial role in facilitating the function of DNA2 in stalled replication fork processing and in DSB end resection. In response to replication stress in fission yeast, Dna2 is phosphorylated by Cds1 at the S220 residue (143). S220 phosphorylation of Dna2 promotes its recruitment to stalled replication forks, as well as the cleavage of nascent strands to prevent the formation of potentially deleterious regressed fork structures (143). Indeed, mutation of S220 causes the dissociation of DNA2 from chromatin after DNA damage. Meanwhile, *sc*DNA2 is phosphorylated at Thr4, Ser17, and Ser237 residues by Cdk1 in response to DSBs. Cdk1-dependent *sc*DNA2 phosphorylation stimulates its localization to the nucleus and to DSB sites (167). *sc*DNA2 is also phosphorylated by the checkpoint kinase Mec1, although the role of this phosphorylation has not been established (167). Proteomic analyses of endogenously ubiquitinated proteins in human cells revealed that DNA2 is ubiquitinated at multiple sites across the protein (169).

It has recently been reported that, in humans, nuclear DNA2 is ubiquitinated. In response to DNA damaging agents, such as camptothecin and hydroxyurea, DNA2 ubiquitination levels increased significantly (170). Furthermore, it was found that the human E3 ligase TRAF6 binds to DNA2 and mediates its K63 ubiquitination. TRAF6-mediated DNA2 ubiquitination promotes its stability and nuclear localization (Figure 5) (170). This TRAF6-mediated spatial regulation mechanism helps to answer the fundamental question of how mammalian DNA2, which lacks an NLS, migrates into nuclei and binds to chromatin during S phase or in response to endogenous or environmental genotoxic stresses. Polyubiquitination of DNA2 may enhance its interaction with ubiquitin-binding domain (UBD)-containing proteins that have an NLS and allow DNA2 to migrate into nuclei via a piggyback transport mechanism (171). Alternatively, the K63-linked ubiquitin chain, which has direct DNA-binding activity (172), may promote DNA2 nuclear localization via an NLS-independent nuclear localization mechanism, similar to that used by the nuclear protein MeCP2 (173). This mechanism of regulation of nuclear localization is quite different from that proposed in yeast, which involves phosphorylation of the N-terminal regulatory region by Cdc28 (homologous to human CDK1 and CDK2) (174). Genetic or chemical inhibition of TRAF6, as well as DNA2 non-ubiquitination mutations, abolished both the ubiquitination and nuclear localization of DNA2 and consequently impaired DNA end resection and HDR of DSBs.

In yeast, *sc*DNA2 is regulated by the SUMOylation of its N-terminal regulatory domain, a region not found in the human enzyme (175). SUMOylation inhibits the nuclease but not the helicase activity of DNA2 *in vitro* and targets DNA2 for degradation *in vivo*. In non-SUMOylatable *dna2* mutants, total Dna2 levels were higher than in strains with SUMOylatable Dna2, but recruitment to nuclei was lower. End resection was also somewhat defective and S phase lengthened in the non-SUMOylatable mutants (175). Thus, DNA2 regulation seems to differ in humans and yeast.

Fe–S cluster assembly can be considered a post-translational modification. The Campbell group has shown that the *sc*DNA2 protein contains an Fe–S cluster domain, consisting of four conserved cysteine residues, C519, C768, C771 and C777, within the nuclease domain (13). Mutation of any of these cysteine residues impairs the nuclease and ATPase activities of *sc*DNA2. This suggests an essential role for the Fe–S cluster in substrate binding and/or catalysis, as well as an interaction between *sc*DNA2 nuclease and helicase activities. Interestingly, biochemical analysis revealed that Cys to Ala mutations that impair these activities do not affect substrate binding affinity but change the substrate binding mode (13). Pokharel and Campbell noticed that the replication-defective yeast strain *dna2-1* harbors a P504S mutation, which is proximal to the Fe–S cluster domain (13). The P504S mutation abolished the nuclease activity of *sc*DNA2, caused temperature sensitivity, and closely mimicked the global defects due to Fe–S cluster mutations (13). Thus, it is likely that the conserved P504 residue is important in stabilizing the Fe–S cluster. The crystal structure of mouse DNA2 bound to ssDNA revealed that the Fe–S cluster is conserved in mammalian DNA2 (35). The structure also indicated that the Fe–S cluster supports the formation of the central tunnel for DNA substrate binding and threading (35), explaining the observation that mutations at the cluster domain alter the DNA substrate binding mode.

## DNA2 IN HUMAN DISEASE

### DNA2 as a tumor suppressor

Considering its importance in multiple DNA metabolic pathways, DNA2 nuclease/helicase is crucial for maintaining genome integrity. In mammalian cells, DNA2 is required to counteract various forms of DNA replication stress; hence, DNA2 functional deficiency in mammalian cells has been found to cause various forms of genome instability. It has long been hypothesized that cancer cells arise and progress due to the accumulation of genetic and epigenetic alterations (176). Consistent with this hypothesis, heterozygous *Dna2* knockout mice have relatively high spontaneous cancer incidence compared to wild-type mice (75). This result suggests that mammalian DNA2 functions as a tumor suppressor by maintaining genome integrity.

One important function of mammalian DNA2 is to facilitate DNA replication at telomeres and centromeres, and its dysfunction at these critical structures may promote the development of cancer. In mouse cells, as originally discovered in yeast (55), heterozygous knockout of the *Dna2* gene resulted in fragile telomeres, telomere shortening, and telomeric sister chromatid exchange (21). Deletion of DNA2 genes in human HCT116 cells resulted in incomplete DNA replication at centromeres, causing dysfunctional centromeres and chromosome segregation errors that led to aneuploidy (77), a hallmark of many human cancers that facilitates cancer development (177). Depletion of DNA2 in human U2OS cells also resulted in the incomplete DNA replication and an increase in the levels of micronuclei and abnormal chromosomes (74). Furthermore, DNA2 deficiency in human cells results in defective processing of stalled replication forks caused by exogenous DNA insults, leading to the accumulation of DSBs, which have been linked to chromosome deletions and translocations (126). In addition, in the absence of DNA2, alternative pathways are employed by cells to cleave DNA flaps and nascent DNA at stalled replication forks. These pathways may release DNA fragments (178) that can be inserted at DSB sites, which is a common occurrence in cancer. Indeed, consistent with the enzymatic and cell biological studies described above, Ira and colleagues have documented the role of *sc*DNA2 in limiting the insertion of retrotransposons or other DNA fragments into DSBs (179). Additional studies are required to determine if DNA2 is critical in suppressing such genome-threatening events in mammalian cells.

DNA2 deficiency and *DNA2* mutations have been linked to human cancers, including gastric cancer (180). Goldberg and colleagues investigated *DNA2* mutations in estrogen-relevant human cancers and reveal that 0.92%, 0.59% and 6.05% of ovarian, breast, and uterine cancers, respectively, carry *DNA2* mutations (181). Most of these somatic *DNA2* mutations are missense mutations and are clustered in the nuclease and helicase domains of DNA2 (181). To comprehensively define somatic *DNA2* mutations in different human cancers, we surveyed a collection of 56 993 specimens from 194 studies for *DNA2* mutations using the cBioportal database (www.cBioportal.org) (182). We identified 280 loss-of-function (frame-shift or splicing) and missense mutations in the *DNA2* gene. *DNA2* mutations were frequent in uterine carcinoma (40/529 or 7.56%), stomach carcinoma (34/999 or 3.5%), bladder cancer (10/411 or 2.43%), and melanoma (10/448 or 2.2%). On the other hand, *DNA2* mutations were significantly less frequent in prostate cancer (11/3647 or 0.3%), chronic lymphocytic leukemia (1/506 or 0.2%), clear cell renal cell carcinoma (1/502 or 0.2%), and low grade glioma (1/514 or 0.19%). Most strikingly, the recurrent *DNA2* frame-shift mutation S779fs*6 occurred in 18 stomach carcinomas, two uterine carcinomas, one adrenocortical carcinoma, and one colorectal carcinoma. Other recurrent mutations included loss-of-function and missense mutations in the nuclease and helicase domains of DNA2. The frequent occurrence of somatic *DNA2* mutations in human cancers is consistent with its role in genome stability and tumor suppression. All these data support a role for DNA2 in cancer etiology and indicate that *DNA2* mutations and functional deficiency may serve to promote cancer development.

### DNA2 as a promoter of cancer progression and potential anti-cancer target

Although DNA2 is crucial for maintaining genome integrity and suppressing neoplastic transformation in normal mammalian cells, it has also been found to support cancer cell survival and progression by counteracting intrinsic and external DNA replication stresses (23,75,183). DNA2 overexpression has been found in human cancers, including breast and pancreatic cancers, and high levels of DNA2 expression have been associated with poor prognosis (23). Due to the critical roles of DNA2 in DNA replication and DSB repair, it has been proposed as an ideal target to sensitize cancer cells to chemotherapy or radiotherapy treatment.

Most conventional chemotherapy agents target replication forks, including those that induce DNA lesions, such as cisplatin, and those that stall forks, such as gemcitabine and 5-fluorouracil (184). In addition, radiotherapy, which is used to treat ∼50% of all cancers, kills cancer cells by inducing DNA damage (185). Thus, inhibiting specific DNA replication and repair proteins like DNA2 has been an attractive anti-cancer strategy. Complete inactivation of either the DNA2 helicase or nuclease is lethal to cells across species, from yeast to humans (7,15,21,25,47,48,74,124,126). Furthermore, as discussed above, DNA2 mutations have been linked to cancers, as well as developmental and mitochondrial disorders. However, humans and mice heterozygous for DNA2 null mutations are viable, due to partially compensating pathways, suggesting that a therapeutic window can be found for DNA2 inhibition, particularly in cancers that overexpress DNA2.

DNA2 plays three key roles at DNA replication forks to enable cancer cells to counteract intrinsic and external DNA replication stresses induced by chemotherapy or radiotherapy: 1) flap removal during DNA replication (11,32,34,46,54,107); 2) stabilization and resolution of reversed forks (76,126,143); and 3) DNA end resection (118,121,122,127,144). DNA2 also acts in signaling, as both an activator and a target of checkpoint kinases. For example, an N-terminal motif in *sc*DNA2 is required to activate the yeast master signaling kinase MEC1/ATR (186). DNA2 is also a target of checkpoint effector kinases CHK1 and CHK2 and is required to regulate potentially deleterious fork reversal and template switching during replication fork stalling in yeast and humans (143,187). Thus, inhibiting DNA2 may simultaneously impair the ability of cancer cells to handle DNA replication stress and DSBs, leading to apoptosis and cellular senescence.

Supporting this hypothesis, recently developed small molecule DNA2 inhibitors have been shown to kill and sensitize cancer cells to ionizing radiation and camptothecin (75,183). A joint effort by the Campbell and Shen groups led to the identification of 4-hydroxy-8-nitroquinoline-3-carboxylic acid (C5), via a high throughput virtual screening, as an effective and selective inhibitor of DNA2 (75). C5 targets a DNA-binding motif in DNA2, blocking substrate binding and inhibiting both its nuclease and helicase activities. As a consequence, C5 inhibits DNA2-mediated resection at stalled forks and at DSBs. C5 is an even more potent inhibitor of stalled DNA replication fork restart and of over-resection of nascent DNA in cells defective in replication fork protection, including those with mutations in *BRCA2*, *RAD51* and *BOD1L*. Notably, DNA2 inhibitors also show cancer cell killing effects that are synergistic with PARP inhibitors (75), which have excited the field as they are synthetic lethal with BRCA1 and BRCA2 deficiencies (188,189). Because PARP is also required to process some Okazaki fragments (73), defects caused by DNA2 inhibitors in either Okazaki fragment processing or HDR may contribute to their synthetic lethality. The Sung, Ira and Peng groups also worked together to screen *sc*DNA2 inhibitors using a fluorescence-based DNA2 nuclease activity assay system (183). They found that compound NSC-105808 selectively inhibits *sc*DNA2 *in vitro*, as well as human DNA2 *in vitro* and in cells. Similar to C5, NSC-105808 reduced DNA end resection and HDR efficiency and sensitized cells to oncogene-induced replication stresses (183).

### DNA2 in mitochondria-based diseases

The environment within mitochondria is known to be highly oxidative. Thus, a robust BER mechanism is required in mitochondria to repair base damage due to reactive oxidative species. The long-patch BER process is critical for efficiently repairing base damage on mtDNA (190,191). DNA2 can cleave nicks or short flaps with 5′ apurinic groups and is required for BER *in vitro* (14). Thus, *DNA2* gene deficiency causes cells to accumulate base damage on mtDNA (14,162) and may lead to various mitochondria-based diseases. A family-based whole genome DNA sequencing study detected the *DNA2* mutation R284H in two siblings with muscle mitochondrial dysfunction but not in their healthy brother (192). In the same study, a *DNA2* mutation screening in 44 patients with mitochondrial myopathy identified two carrying K313E and V723L *DNA2* mutations, respectively. Biochemical analyses revealed that these mutations altered the nuclease and helicase activities of DNA2. Furthermore, DNA2 mutant muscular tissues, similar to Pol γ and Twinkle mutant muscular tissues, displayed multiple mtDNA deletions (192,193). Another study identified a germline *DNA2* truncation mutation (Asn568Ilefs*4), which eliminates the ATPase and helicase domains of human DNA2, in a patient who displayed congenital myopathy and ptosis (194). More recently, four additional germline *DNA2* mutations, A221G, S552L, S640L, R959H, were identified in patients with myopathy (195). These mutations significantly reduced the nuclease, ATPase, and helicase activities of DNA2 in biochemical assays *in vitro*. These findings suggest a role of *DNA2* mutations in the pathogenesis of human mitochondrial disorders.

### DNA2 in primordial dwarfism (PD)

In addition to mitochondrial myopathy and human cancer, DNA2 has been implicated in PD. Patients with PD have stunted growth, resulting in small adult body size, due to severe impairments in fetal growth and postnatal development (89,196). Seckel syndrome, a type of PD, has been linked to abnormal centrosome assembly and DNA damage responses (197). Autozygome-guided mutation analysis detected a c.3372 + 6delC germline mutation shared by two distant relatives within a family affected by PD (89). The mutation causes DNA2 protein truncation and a reduction in its gene expression, thus leading to its functional deficiency (89). In another study, whole exosome sequencing of 192 patients with microcephalic PD identified variants in the DNA2 gene: two intronic variants (c.1764-38_1764-37ins(53) and c.74+4A>C) and a missense variant (c.1963A>G, T655A) (196). The authors demonstrated that the two intronic variants alter DNA2 transcript splicing. The T655A occurs at the conserved residue within the ATP binding motif. These studies suggest that DNA2 is a PD gene.

## CONCLUDING REMARKS

DNA2 nuclease/helicase is a multifunctional enzyme that plays critical roles in various DNA metabolic pathways, including Okazaki fragment maturation, centromeric DNA replication, maintenance of telomere stability, stalled replication fork processing, and HDR. The many, often redundant activities of DNA2 have made it difficult to dissect its precise molecular functions in each process. Though much progress has been made, especially in understanding the role of DNA2 in flap processing, we still do not understand why the long-flap pathway evolved and whether it functions in the removal of errors made by Pol α/primase. The contribution of DNA2 to Okazaki fragment processing in metazoans also remains ripe for exploration. Future studies to understand the functions and regulation of DNA2 in the resection of DSBs, as well as its contributions to pathway choice, checkpoint activation, and HDR, will require careful, stepwise biochemical reconstitution experiments combined with genetic approaches, similar to those applied to investigate its contribution to lagging strand DNA replication. Future studies will also need to address the major question of which types of lesions require which resection pathways and how the multiple pathways are coordinated and regulated. Answering these questions is also required to delineate the mechanisms underlying the role of DNA2 at stalled DNA replication forks and in replication restart. DNA2 also plays a direct role in protein-protein interactions that induce the activity of the Mec1 kinase and the DNA damage response in yeast. Whether metazoan DNA2, in addition to ATRIP and ETAA1, also performs this role in either constitutive or exogenous DNA damage-induced checkpoints has not been demonstrated.

Interestingly, DNA2 functions as both a tumor suppressor and promoter. In normal cells, it works with other genome stability genes to maintain the integrity of the genome and to avoid neoplastic transformation. However, DNA2-mediated pathways that counteract replication stresses and repair DSBs are also utilized to promote cancer cell survival. Therefore, although, like other current chemotherapeutics, inhibitors of DNA2 may potentially initiate new cancers, DNA2 has been suggested as a cancer therapeutic target. Given that many cancers are repair-defective, whereas normal cells contain intact repair pathways, there is hope that a therapeutic window may be found to kill cancers while sparing healthy tissues.
